# Habitat suitability assessment of wild medicinal plant resources in the Changbai Mountain ecosystem of China Using MaxEnt and AHP

**DOI:** 10.3389/fpls.2026.1776217

**Published:** 2026-04-01

**Authors:** Xiaohui Li, Xiaoqing Tang, Cuicui Ren, Ge Yan, Zhanbo Li, Zhonghua Tang, Liqiu Zhang, Ying Liu, Dewen Li

**Affiliations:** 1 Key Laboratory of Forest Plant Ecology, Ministry of Education, Northeast Forestry University, Harbin, Heilongjiang, China; 2 College of Food and Health, Northeast Forestry University, Harbin, Heilongjiang, China; 3 Key Laboratory of Ecological Utilization of Forest Source Active Substances, Northeast Forestry University, Harbin, Heilongjiang, China; 4 College of Medicine, Tonghua Normal University, Tonghua, Jilin, China

**Keywords:** analytic hierarchy process (AHP), bioclimatic variables, Changbai Mountain ecosystem, maximum entropy (MaxEnt), suitable habitat, wild medicinal plants

## Abstract

**Introduction:**

Plants have been an essential source of Chinese traditional medicine for millennia, and there are more wild medicinal plant resources in Changbai Mountain ecosystem. However, due to multiple factors such as habitat changes, the wild medicinal plant resources are facing disadvantage situations, and their sustainable utilization is being restricted.

**Methods:**

In the study, 224 wild medicinal plants were analyzed by analytic hierarchy process (AHP) to make the priority resources for development and utilization. Multi-source species distribution data and bioclimatic variables were utilized to predict the potential habitat suitability of medicinal plant resources and their response to future climate scenarios using Maximum Entropy (MaxEnt).

**Results:**

The results showed that under the climate scenario at present, these wild medicinal plants were classified into three classes based on comprehensive assessments, such as Class I (39 species), Class II (49 species), Class III plant resources (136 species), the suitable habitats for Class I was the largest. The prediction accuracy was evaluated by area under curve (AUC), true skill statistic (TSS) and Cohen’s kappa statistic (KAPPA), respectively above 0.8, 0.85 and 0.75. Under different climate scenarios in 2090s, the suitable habitats for Class II were increased, with the largest suitable habitats reaching 3.61×10⁴ km² by SSP5-8.5, and the centroid of suitable habitats migrated northwestward and northeastward in Jilin Province, with the maximum displacement reaching 27.24 km by SSP2-4.5. Key climatic variables were identified by the jackknife test within the MaxEnt model as the mean temperature of the coldest quarter (Bio11) and annual precipitation (Bio12).

**Discussion:**

Therefore, exploitable potential of Class II plant resources was predicted to surpass Class I in the future. It was a scientific basis for integrated models to promote the sustainable development of natural resources in mountain ecosystems globally.

## 1 Introduction

Medicinal plants are used to maintain physical, mental, and spiritual health in all cultures and in a variety of capacities and contexts (Davis and Choisy, 2024). As a region rich in wild medicinal plant resources, the Changbai Mountain ecosystem in Jilin Province, spanning 127°42′55″ to 128°16′48″ east longitude and 41°41′49″ to 42°25′18″ north latitude, is characterized by its cold-temperate climate and exceptionally high forest coverage, having a rich diversity of wild medicinal plants (Zhang et al., 2022). Changbai Mountain is located in the cold temperate zone. It has a cold climate and complex terrain, with an extremely high forest coverage rate. These unique natural conditions have jointly given rise to an extremely rich resource of wild medicinal plants (Zhou, 2022; Zhao et al., 2011). Despite their ecological and economic significance, there remains a critical lack of public awareness regarding the conservation status of these medicinal plants. Due to excessive exploitation, the wild medicinal plant resources in this area have significantly decreased (Zhang et al., 2014). Climate change is one of the main drivers of biodiversity loss. Extreme climate events - such as extreme droughts, record high temperatures, and heat waves - will further exacerbate the risk of species decline and even extinction (Isbell et al., 2015). In this context, the lower the biodiversity, the weaker the ecosystem’s resistance to climate stress, and thus the study of wild medicinal plant resources in Changbai Mountain under future climate change scenarios becomes particularly important. Thus, the balance between the growing demand for medicinal plant resources and the necessity of conservation must be maintained to prevent further biodiversity loss (Shao, 2011). This study evaluated the development levels of wild medicinal plant resources, identified the dominant climatic driving factors and changes in the suitable habitats. The relevant results can provide scientific support for the sustainable utilization of wild medicinal plant resources in Changbai Mountain, and also contribute to the long-term protection of biodiversity.

Against the backdrop of intensifying global climate change and persistent anthropogenic disturbances to natural ecosystems—including population growth, advancements in agricultural technology, and human exploitation of wild medicinal plant resources—the ecological distribution patterns of wild medicinal plant resources are undergoing significant transformations (Nungula et al., 2024). Now, a system integrating the Analytic Hierarchy Process (AHP) and Maximum Entropy (MaxEnt) provides a solid framework for potential exploitation assessment of medicinal plants, and has been widely used (Arulbalaji et al., 2019; Mustafa and Mehmet, 2023). The AHP has been demonstrated its utility in evaluating landscape restoration strategies for community parks (Cheng and Li, 2025), quantified the ecological and ornamental value of native rhododendrons (Liang et al., 2024), and identified key genotype for sustainable potato cultivation (Liang et al., 2023). In particular, the MaxEnt has become a powerful approach for predicting distributions under future climatic scenarios (Yalcin et al., 2023). MaxEnt has been widely applied to predict species habitat distributions and assess the impacts of current and future climatic conditions on species. For instance, in the study by Malakar et al., the impact of climate change on *A. indica* was predicted by modeling its probable habitat distribution under current and future climatic scenarios using MaxEnt under four different Shared Socioeconomic Pathways (Malakar et al., 2025). Similarly, Garai et al. utilized the same approach to identify the most suitable climatic zones and key environmental variables for species survival and optimal proliferation (Garai et al., 2025). Furthermore, MaxEnt has also been applied to evaluate the response of vulnerable species such as *Buchanania cochinchinensis* to climate change (Garai et al., 2023). These models provide a comprehensive system to analyze the development potential of wild medicinal resources in the Changbai Mountain ecosystem under climatic scenarios of the future and the present.

This study fills the previous research gap and is novel in two key aspects: Firstly, it combines the qualitative assessment of the development potential of wild medicinal plant resources with habitat suitability prediction, overcoming the limitations of previous single-focused studies; Secondly, it predicts the habitat dynamics under various future climate scenarios (SSP1-2.6, SSP2-4.5, SSP3-7.0, SSP5-8.5), providing forward-looking support for adaptive conservation strategies. The main objectives of this study are: (1) To assess the development level of wild medicinal plant resources in Changbai Mountain under the current climate scenario; (2) To determine the main bioclimatic driving factors affecting the distribution of wild medicinal plant resources; (3) To simulate the dynamic changes of suitable habitats for wild medicinal plant resources under future climate scenarios; (4) To provide scientific support for the sustainable utilization of wild medicinal plant resources in the Changbai Mountain ecosystem and the long-term conservation of biodiversity.

## 2 Materials and methods

### 2.1 Distribution of wild medicinal plants in Changbai Mountain

Field investigations were conducted in 12 representative ecological regions (Antu, Baishan, Fu Song, Linjiang, Liuhe, Tonghua, Jiutai, Changchun, Jilin, Yitong, Dunhua and Wangqing) to document the wild medicinal plant resources. The species distribution data were obtained from the Fundamental Research Funds for the Central Universitie (2572023CT11). The investigation employed a grid-based sampling method with grid cell dimensions of 8 km by 8 km. Within each grid, standard plots were set up based on vegetation types: arbors were 20 m by 20 m, shrubs were 5 m by 5 m, and herbs were 1 m by 1 m. The sampling rate in the total study area was 32%, and all distribution records were collected from these field plots. Through comprehensive records, this study identified 224 medicinal plants, representing 56 plant families, and detailed characteristics are described in the **Supplementary Materials** (**Table A.1**).

**Figure 1:**
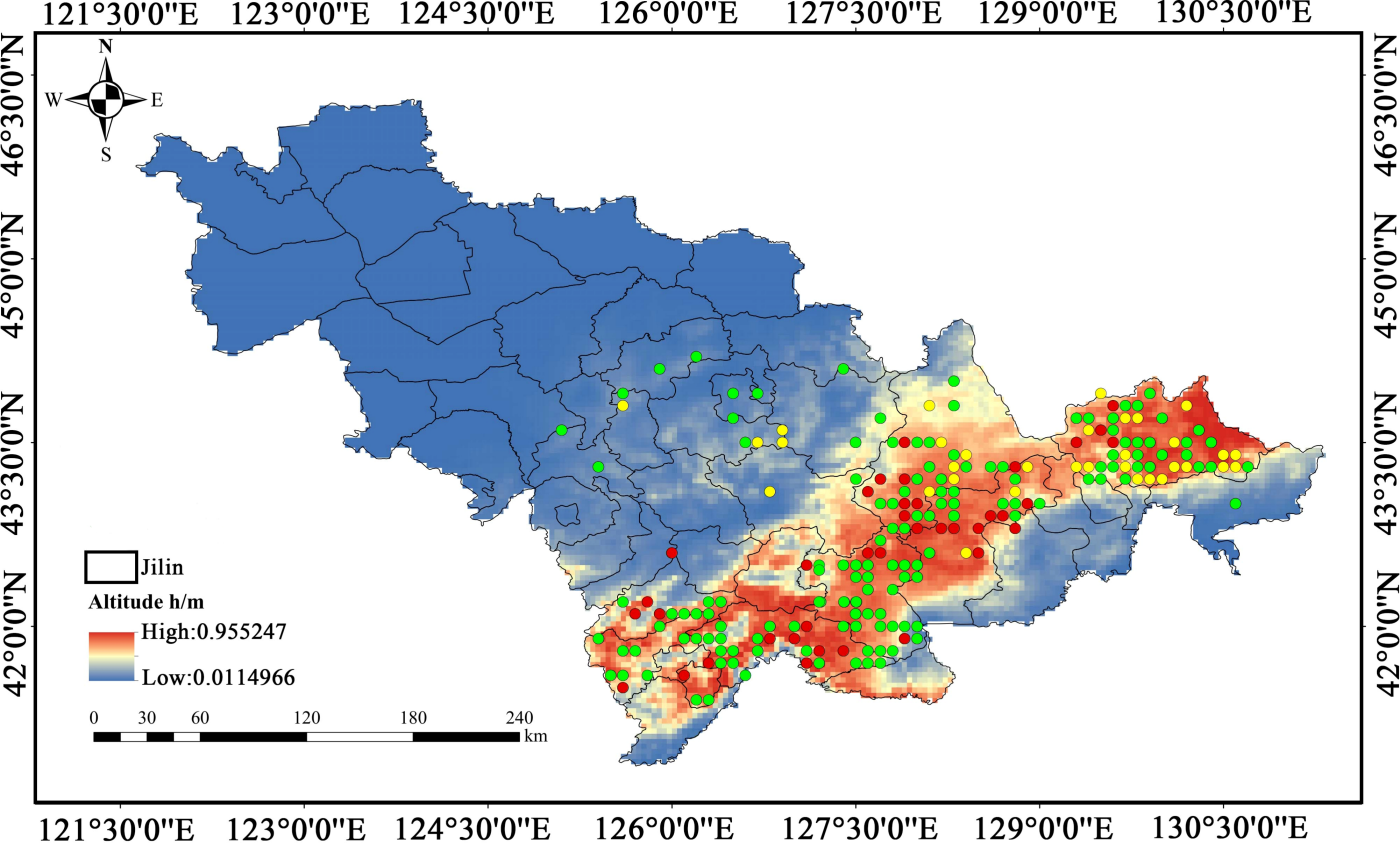
Distribution sites of medicinal plant resources in Changbai Mountain ecosystem from Jilin province.

The geospatial framework for analysis was constructed using the official base map obtained from the Standard Map Service Platform (http://bzdt.ch.mnr.gov.cn/) administered by the Ministry of Natural Resources of China. The spatial distribution of these resources was presented in the accompanying map (**Figure 1**).

### 2.2 Environmental variables used in MaxEnt

The bioclimatic variable data are sourced from the WorldClim database (http://www.worldclim.org/). These variables were generated through spatial interpolation of long-term observational data from global weather stations and were further processed to reflect annual trends, seasonal variations, and extreme or restrictive climatic factors. Under the historical climate scenario (1970 to 2000), a total of 19 bioclimatic variables were downloaded for this study (**Table 1**). IBM SPSS 21 software was used to analyze the correlation of climate variable data, and combined with the matrix of contribution rate and correlation coefficient, Pearson correlation coefficient (r) was used to test the multicollinearity among variables, and the bioclimatic variables with low contribution rate and correlation coefficient |r| > 0.8 were eliminated (Famiglietti et al., 2023). The jackknife tests was used to test and evaluate the contribution rate of each bioclimatic variable to the model. By sequentially eliminating individual variables and monitoring the changes in model performance, the importance of each environmental variable was assessed, thereby quantifying the relative contribution of each bioclimatic variable to the final MaxEnt model (Esfanjani et al., 2018).

**Table 1:** Bioclimatic variables.

Variables	Variable name	Unit
Bio1	Annual mean temperature	°C
Bio2	Mean diurnal temperature range	°C
Bio3	Isothermality	–
Bio4	Temperature seasonality	°C
Bio5	Maximum temperature of warmest month	°C
Bio6	Minimum temperature of coldest month	°C
Bio7	Temperature annual range	°C
Bio8	Mean temperature of wettest quarter	°C
Bio9	Mean temperature of driest quarter	°C
Bio10	Mean temperature of warmest quarter	°C
Bio11	Mean temperature of coldest quarter	°C
Bio12	Annual precipitation	mm
Bio13	Precipitation of wettest month	mm
Bio14	Precipitation of driest month	mm
Bio15	Precipitation seasonality	–
Bio16	Precipitation of wettest quarter	mm
Bio17	Precipitation of driest quarter	mm
Bio18	Precipitation of warmest quarter	mm
Bio19	Precipitation of coldest quarter	mm

### 2.3 AHP and MaxEnt modelling process

**Figure 2:**
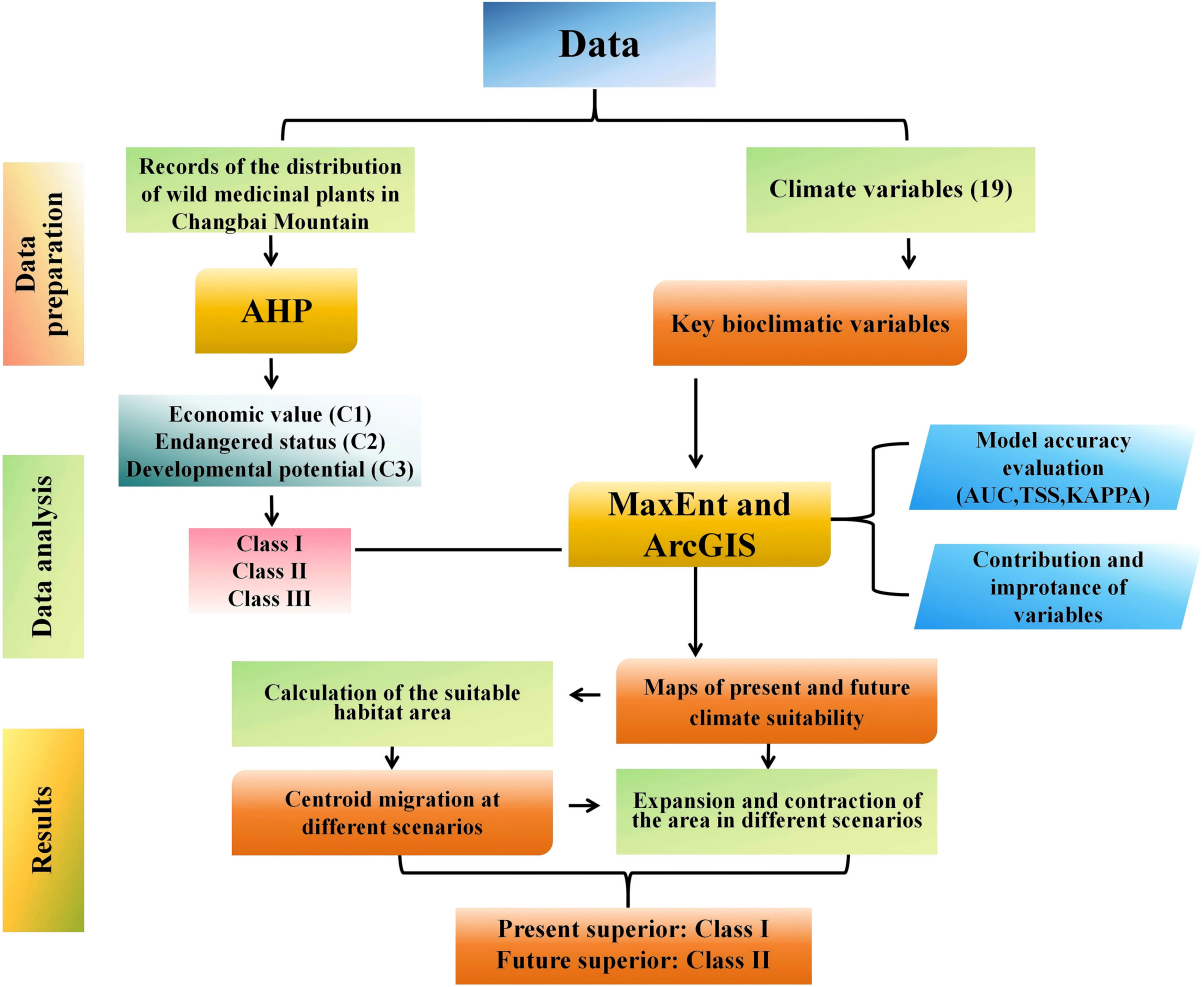
AHP–MaxEnt models method.

In the study, the experimental design was shown in **Figure 2**. The AHP was composed of the target layer, criterion layer, and indicator layer. The relative importance weights of each criterion were quantified using Saaty’s 1–9 scale through yaahp10.3 software, ensuring a consistency ratio (CR) below 0.1. This process yielded a prioritized ranking of indicator significance. MaxEnt (v.3.4.4) is a machine learning model based on the principle of maximum entropy. It estimates the potential geographical distribution of species solely using presence records and environmental variables. It has stable performance and high prediction accuracy, and was highly applicable to limited occurrence data. Therefore, it has been widely used in species distribution modeling. In this study, 75% of the occurrence points were randomly selected for model training, and 25% were used for testing the prediction accuracy. The model underwent 10 cross-validations and the average results were adopted.

The 224 wild medicinal plant species were classified into three grades based on comprehensive scores: Class I (> 2.2000, 39 species), Class II (2.1000–2.2000, 49 species), and Class III (< 2.1000, 136 species). Based on the output of the AHP, the suitable habitats for wild medicinal plants were quantified by using MaxEnt 3.4.1 model and ArcGIS 10.6. Three independent MaxEnt models were separately constructed based on the occurrence records of each grade. Based on the suitability threshold predicted by the MaxEnt model, the habitat suitability index was classified into four levels using the natural breakpoint method (Kang et al., 2025). The potential suitable areas were classified into four levels: non-suitable (0 - 0.2), low-suitable (0.2 - 0.4), moderately-suitable (0.4 - 0.6), and highly-suitable (> 0.6). To assess the impacts of future climate change, four contrasting climate scenarios from CMIP6 were employed in this study: SSP 1-2.6 (sustainable development with low greenhouse gas forcing), SSP 2-4.5 (moderate development with medium forcing), SSP 3-7.0 (regional rivalry with high forcing), and SSP 5-8.5 (fossil-fueled development with very high forcing). These scenarios represent distinct socioeconomic development pathways and radiative forcing levels into the future. The average distribution center of wild medicinal plants in the Changbai Mountain ecosystem was identified under present and future climate scenarios and migration characters were displayed.

### 2.4 Verification of MaxEnt model accuracy

The distribution point data of wild medicinal plants and bioclimatic variables were cross-verified by MaxEnt model for 10 times and the average value was taken. Of all occurrence points, 75% were randomly used for model training, and 25% for testing the prediction accuracy. The regularization multiplier is set to the default value of 1.0, and the default model features (linear, quadratic, product, threshold, and hinge) are applied. The maximum number of iterations is set to 5000, and the convergence threshold is set to 10^-5^. A total of 10,000 background points are randomly generated for model training. The area under curve (AUC), true skill statistic (TSS) and Cohen’s kappa statistic (KAPPA) were used to comprehensively evaluate the accuracy of Maxent the values were 0.5-1, respectively; the ROC curve was used a crucial evaluation tool that reflected the trade-off relationship between the true positive rate (sensitivity) and the false positive rate (1-specificity) of the model under different decision thresholds (Li et al., 2022).

## 3 Result

### 3.1 Model based on AHP

#### 3.1.1 Formulation of pairwise comparison matrices

Four judgment matrices were constructed using yaahp software: A - C3, C1 - P5, C2 - P12, and C3 - P20, with corresponding maximum eigenvalues: λmax1 = 3.0537, λmax2 = 5.4027, λmax3 = 7.7351, λmax4 = 8.9483. Validation showed that the Consistency Ratios (CR) for these matrices were CR1 = 0.0517, CR2 = 0.0899, CR3 = 0.0901, and CR4 = 0.0961 (All less than 0.1). This confirmed consistency across all matrices, thereby validating the derived indicator weights for hierarchical decision-making (**Tables 2**, **3**).

**Table 2:** Judgment matrix of Target layer (A) and constraint layer (C).

A	C_1_	C_2_	C_3_	W_i_
C_1_	1	2	1/2	0.3119
C_2_	1/2	1	1/2	0.1976
C_3_	2	2	1	0.4905

W_i_ denotes the weight value in the table; the same applies hereinafter.

**Table 3:** Judgment matrix of constraint layer (C) and standard layer (P).

C_1_	P_1_	P_2_	P_3_	P_4_	P_5_	P_6_	P_7_	P_8_	W_i_
P_1_	1	1/4	1/3	1/2	1/2				0.0860
P_2_	4	1	2	1/3	2				0.2425
P_3_	3	1/2	1	1/2	3				0.2050
P_4_	2	3	2	1	3				0.3564
P_5_	2	1/2	1/3	1/3	1				0.1102
C_2_	P_6_	P_7_	P_8_	P_9_	P_10_	P_11_	P_12_		
P_6_	1	2	3	1/2	2	2	1/2		0.1595
P_7_	1/2	1	2	1/2	2	1/3	2		0.1372
P_8_	1/3	1/2	1	1/2	2	1/2	1/4		0.0757
P_9_	2	2	2	1	2	1/2	2		0.1907
P_10_	1/2	1/2	1/2	1/2	1	1/3	1/2		0.0645
P_11_	1/2	3	2	2	3	1	1		0.2073
P_12_	2	1/2	4	1/2	2	1	1		0.1651
C_3_	P_13_	P_14_	P_15_	P_16_	P_17_	P_18_	P_19_	P_20_	
P_13_	1	1/2	1/3	1/3	2	2	1/2	2	0.0949
P_14_	2	1	2	2	3	2	3	2	0.2188
P_15_	3	1/2	1	3	4	2	3	3	0.2177
P_16_	3	1/2	1/3	1	3	1	1/2	2	0.1207
P_17_	1/2	1/3	1/4	1/3	1	2	1	1/3	0.0667
P_18_	1/2	1/2	1/2	1	1/2	1	1	1/2	0.0744
P_19_	2	1/3	1/3	2	1	1	1	3	0.1188
P_20_	1/2	1/2	1/3	1/2	3	2	1/3	1	0.0881

The table comprises three judgment matrices, labeled sequentially as C_1_-P_5_, C_2_-P_12_, and C_3_-P_20_.

#### 3.1.2 Criteria weight prioritization

In the constraint layer system, the order of weights was developmental potential (C3) > economic value (C1) > endangered status (C2). Therefore, developmental potential played a dominant role in the assessment of medicinal plant sustainable utilization (**Table 4**).

**Table 4:** Evaluation model indicator weight determination results and hierarchical total ranking.

Constraint layer weight	Criteria layer	Criteria layer weight	Comprehensive weight	Total ranking
Economic Value (C_1_)	Field Management (P_1_)	0.0860	0.0268	18
	Medicinal Value (P_2_)	0.2425	0.0756	4
	Widely Applicable (P_3_)	0.2050	0.0639	5
	Comprehensive development (P_4_)	0.3564	0.1112	1
	Market Price (P_5_)	0.1102	0.0344	13
Endangered Status (C_2_)	Wild Resource Abundance (P_6_)	0.1595	0.0315	16
	Taxonomic (P_7_)	0.1372	0.0271	17
	Distribution Range (P_8_)	0.0757	0.0150	19
	Distribution Frequency (P_9_)	0.1907	0.0377	11
	Population Density (P_10_)	0.0645	0.0127	20
	Disease Resistance (P_11_)	0.2073	0.0410	10
	Protection Level (P_12_)	0.1651	0.0326	15
Development Potential (C_3_)	Habitat Requirements (P_13_)	0.0949	0.0466	8
	Cultivation Status (P_14_)	0.2188	0.1073	2
	Reproduction Difficulty (P_15_)	0.2177	0.1068	3
	Growth Cycle (P_16_)	0.1207	0.0592	6
	Continuous Cropping Obstacle (P_17_)	0.0667	0.0327	14
	Survival Rate (P_18_)	0.0744	0.0365	12
	Commercial Grade (P_19_)	0.1188	0.0582	7
	Planting Density (P_20_)	0.0881	0.0432	9

#### 3.1.3 Classification level of wild medicinal plants in Changbai Mountain

Based on comprehensive assessment values derived from weighted scores, three classifications were established: Class I (> 2.2000), Class II (2.1000 - 2.2000), and Class III (< 2.1000). Utilizing this model, 224 wild medicinal plant resources in Changbai Mountain ecosystem were categorized into distinct sustainability levels (**Table 5**), with detailed outcomes provided in **Supplementary Materials** (**Table A.2**).

**Table 5:** Total ranking of wild medicinal plants in Changbai Mountain.

Class	Species name	Integrated assessment value
Class I (> 2.2000)	*Elsholtzia ciliata*	2.6029
	*Taraxacum mongolicum*	2.5771
	*Commelina communis*	2.5331
	*Plantago depressa*	2.524
	*Asarum heterotropoides*	2.4536
	*Sanguisorba officinalis*	2.4158
	*Equisetum hyemale*	2.3912
	*Adenophora tetraphylla*	2.3843
	*Scutellaria baicalensis*	2.3768
	*Campanula punctata*	2.3517
	*Aconitum kusnezoffii*	2.3505
	*Viola acuminata*	2.3475
	*Smilax riparia*	2.3257
	*Bidens parviflora*	2.2985
	*Plantago asiatica*	2.2933
	*Patrinia scabiosaefolia*	2.293
	*Pseudostellaria heterophylla*	2.293
	*Vicia unijuga*	2.2842
	*Panax ginseng*	2.2808
	*Ligularia fischeri*	2.276
	*Potentilla chinensis*	2.2745
	*Codonopsis lanceolata*	2.271
	*Chloranthus japonicus*	2.2698
	*Polygonatum odoratum*	2.2656
	*Aster tataricus*	2.2653
	*Vicia ramuliflora*	2.2484
	*Euphorbia fischeriana*	2.2462
	*Leibnitzia anandria*	2.237
	*Scutellaria pekinensis*	2.2357
	*Lathyrus davidii*	2.2278
	*Chenopodium album*	2.2204
	*Bupleurum chinense*	2.2192
	*Platycodon grandiflorus*	2.2181
	*Inula japonica*	2.214
	*Ostericum maximowiczii*	2.2116
	*Ranunculus japonicus*	2.2079
	*Dioscorea nipponica*	2.207
	*Solidago decurrens*	2.2036
	*Erigeron annuus*	2.2023
Class II (2.1000 - 2.2000)	*Potentilla cryptotaeniae*	2.1956
	*Menispermum dauricum*	2.1929
	*Sophora flavescens*	2.1928
	*Paris verticillata*	2.1923
	*Rubia cordifolia*	2.191
	*Isodon excisus*	2.1889
	*Isodon japonicus* var. *glaucocalyx*	2.185
	*Vicia pseudorobus*	2.1838
	*Chelidonium majus*	2.1835
	*Arisaema amurense*	2.1835
	*Chamerion angustifolium*	2.1791
	*Ostericum grosseserratum*	2.1771
	*Phryma leptostachya subsp. asiatica*	2.1755
	*Synurus deltoides*	2.1744
	*Cimicifuga dahurica*	2.1707
	*Saposhnikovia divaricata*	2.1686
	*Melampyrum roseum*	2.1671
	*Lycopus lucidus*	2.1659
	*Urtica angustifolia*	2.1594
	*Gentiana scabra*	2.1591
	*Convallaria majalis*	2.1553
	*Astilbe chinensis*	2.1532
	*Artemisia japonica*	2.1532
	*Thalictrum squarrosum*	2.1523
	*Ixeris chinensis*	2.1477
	*Maianthemum japonicum*	2.1449
	*Hemerocallis citrina*	2.1447
	*Lactuca indica*	2.1431
	*Silene fulgensW*	2.1411
	*Geranium wilfordii*	2.1385
	*Aquilegia oxysepala*	2.1368
	*Glechoma longituba*	2.1354
	*Viola prionantha*	2.1351
	*Pyrola rotundifolia*	2.132
	*Angelica dahurica*	2.1319
	*Adenophora trachelioides*	2.1292
	*Rhodiola sachalinensis*	2.1291
	*Iris sanguinea*	2.1257
	*Artemisia argyi*	2.1187
	*Codonopsis pilosula*	2.117
	*Matteuccia struthiopteris*	2.1165
	*Hemerocallis middendorffii*	2.1163
	*Clematis terniflora* var. *mandshurica*	2.1142
Class III (< 2.1000)	*Hylodesmum podocarpum subsp. fallax*	2.0988
	*Astilbe grandis*	2.0958
	*Polygonum thunbergii*	2.0956
	*Cypripedium macranthum*	2.0955
	*Potentilla kleiniana*	2.0952
	*…*	…

### 3.2 Contribution of environmental variables

For Class I plants, the key environmental variables influencing their potential distribution included Bio10 (Mean temperature of warmest quarter), collectively accounting for 52.3% of the total contribution rate. For Class II plants, the key environmental variables influencing their potential distribution included Bio10, collectively accounting for 53% of the total contribution rate. Meanwhile, Class III plants were primarily determined by Bio8 (Mean temperature of wettest quarter), collectively accounting for 55.1% of the total contribution rate. Notably, the common influencing factors of the three kinds of plants were Bio11 and Bio12, which showed that temperature and rainfall were the main influencing factors (**Table 6**).

**Table 6:** Selected bioclimatic Variables for Class I – III Plant resources.

Class	Variable	Percent contribution (%)	Permutation importance (%)
Class I	Bio10	52.3	0.5
	Bio11	2.5	1
	Bio12	2.1	7.3
	Bio13	1.8	1.4
Class II	Bio4	36	24.6
	Bio10	53	0
	Bio11	1.5	0.8
	Bio12	2.1	7.8
	Bio13	2.5	1.5
Class III	Bio4	29.5	9.1
	Bio8	55.1	0
	Bio11	6.6	3.6
	Bio12	2.5	9.3

**Figure 3:**
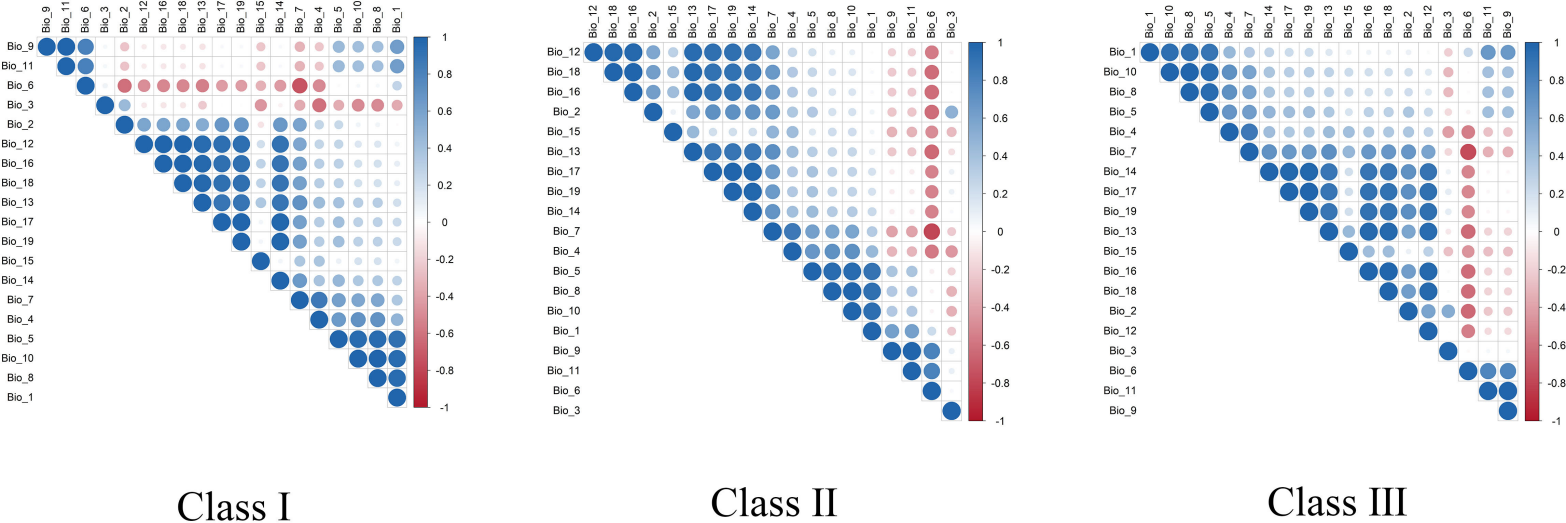
Correlation analysis of bioclimatic variables affecting the distribution of wild medicinal plant resources in Changbai Mountain area.

**Figure 4:**
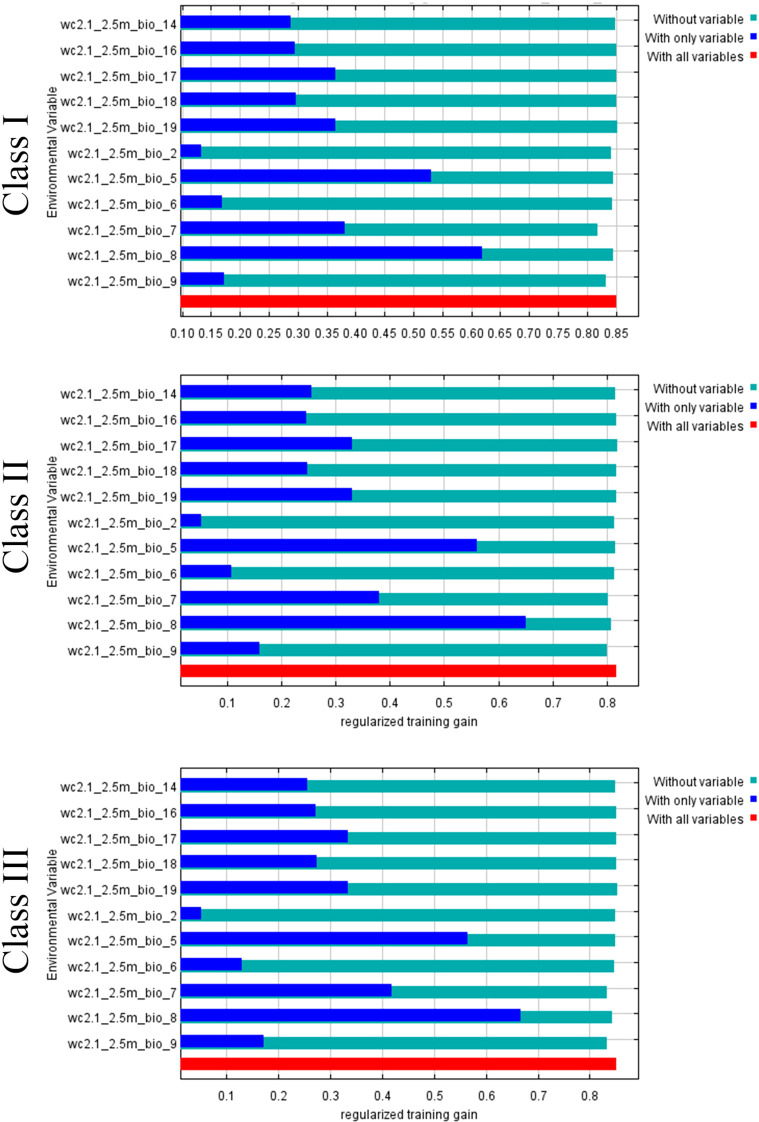
Jackknife test analysis of bioclimatic variables.

In order to avoid the influence of multicollinearity among environmental variables on the accuracy of the model, this study first analyzes the correlation of 19 bioclimatic variables (**Figure 3**). On this basis, the importance of various bioclimatic variables is further evaluated by Jackknife test (**Figure 4**).

### 3.3 Distribution characteristics of suitable habitats for Class I - III plant resources in the climate scenarios at present

In the climate scenario at present, for Class I plants, the highly suitable habitats were 3.48×10^4^ km^2^ (16.37% of Jilin Province’s total area), the moderately suitable habitats were 2.43×10^4^ km^2^ (11.43%) and the low-suitable habitats were 3.65×10^4^ km^2^ (17.17%); For Class II plants, the highly suitable habitats were 3.36×10^4^ km^2^ (15.80%), the moderately suitable habitats were 2.60×10^4^ km^2^ (12.26%) and the low-suitable habitats were 3.73×10^4^ km^2^ (17.54%); For Class III plants, the highly suitable habitats were 3.71×10^4^ km^2^ (17.45%), the moderately suitable habitats were 2.55×10^4^ km^2^ (10.58%) and the low-suitable habitats were 3.33×10^4^ km^2^ (15.66%), respectively.

**Figure 5:**
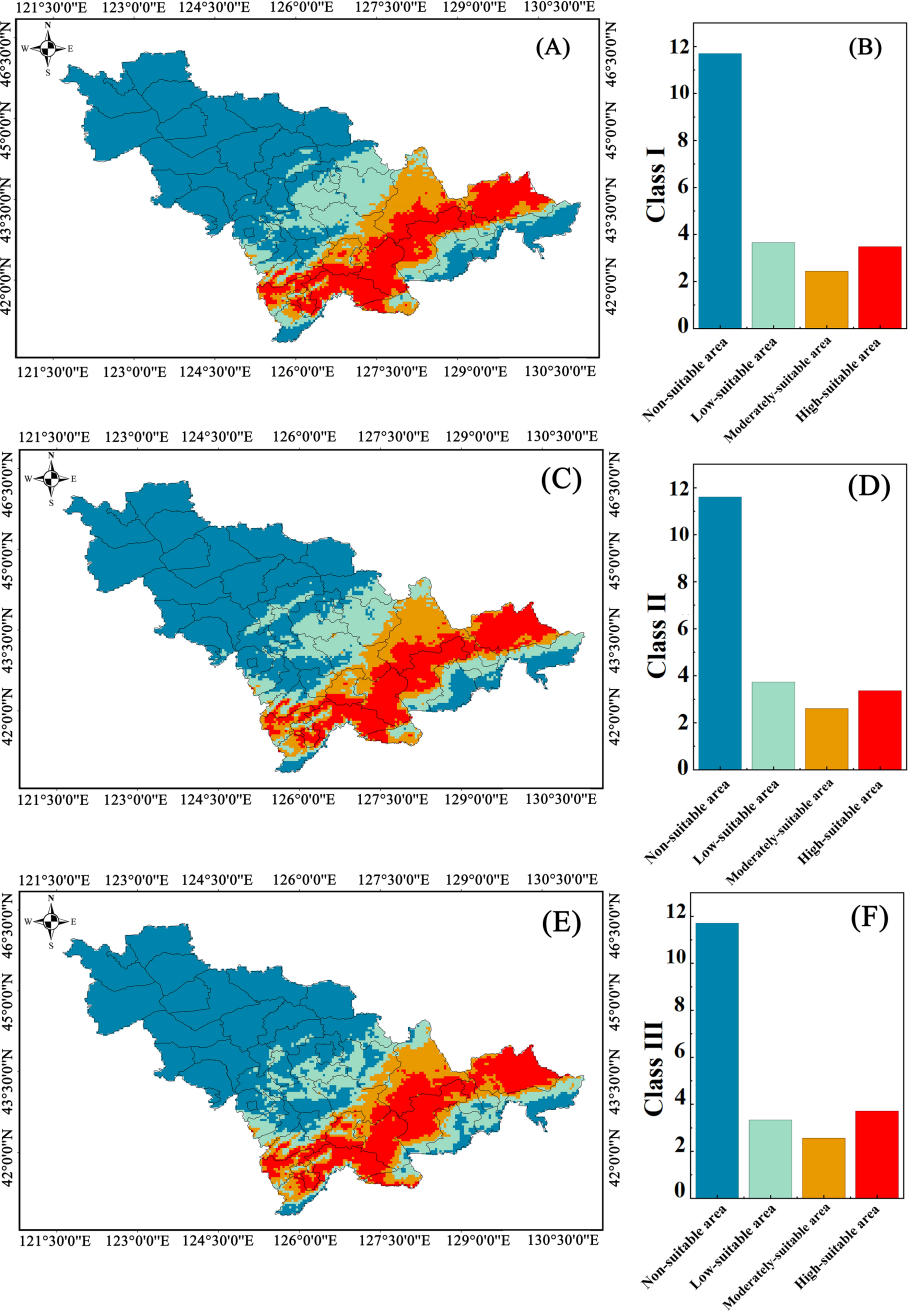
Distribution characteristics of suitable habitat for class I – III plant resources at present. **(A–C)** Diagram of Plant Area Distribution. **(D–F)** The chart represents the area.

The average suitable habitat area was calculated as the sum of low-, moderately, and highly suitable areas (excluding unsuitable areas) divided by the number of species in each class. For Class I plants, the average suitable habitat was 2,451.28 km^2^, while that for Class II and Class III plants was 1,977.55 km^2^ and 705.15 km^2^, respectively (**Figure 5**). The suitable habitat of Class I was significantly larger than that of Classes II and III. Therefore, the AHP-based classification system exhibited clear effectiveness in prioritizing habitat suitability under the present climate scenario.

### 3.4 Distribution characteristics of suitable habitat for Class I – III plant resources in different climate scenarios in 2090s

**Figure 6:**
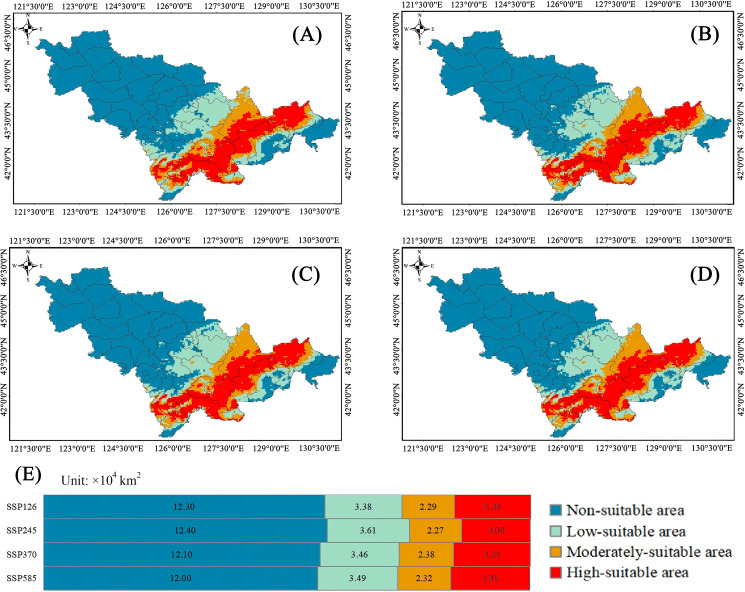
Distribution characteristics of suitable habitat for class I plant resources under future climate scenarios. **(A–D)** Diagram of Plant Area Distribution. **(E)** The chart represents the area.

For Class I plants, in the scenario of SSP 1-2.6 (radiation forcing will reach 2.6 W·m^-2^ in 2100), the highly suitable habitats were 3.38×10^4^ km^2^ (15.89%), the moderately suitable habitats were 2.29×10^4^ km^2^ (10.77%) and the low-suitable habitats were 3.34×10^4^ km^2^ (15.71%). The total suitable areas of Class I plants were about 9.01 × 104 km^2^. Compared with the climate scenario at present, all suitable habitats had been reduced. In the scenario of SSP 2-4.5 (radiation forcing will reach 4.5 W·m^-2^ in 2100), the highly suitable habitats were 3.61×10^4^ km^2^ (16.98%), the moderately suitable habitats were 2.27×10^4^ km^2^ (13.03%) and the low-suitable habitats were 3.00×10^4^ km^2^ (14.11%). Compared with the climate scenario at present, the highly suitable habitats and the moderately suitable habitats increased by 0.61% and 1.6%, respectively. In the scenario of SSP 3-7.0 (radiation forcing will reach 7.0 W·m^−2^ in 2100), the highly suitable habitats were 3.46×10^4^ km^2^ (16.27%), the moderately suitable habitats were 2.38×10^4^ km^2^ (15.76%) and the low-suitable habitats were 3.35×10^4^ km^2^ (14.11%). In the scenario of SSP 5-8.5 (radiation forcing will reach 8.5 W m^−2^ in 2100), the highly suitable habitats were 3.49×10^4^km^2^ (16.42%), the moderately suitable habitats were 2.32×10^4^ km^2^ (10.94%) and the low-suitable habitats were 3.45×10^4^ km^2^ (16.23%). Compared with the climate scenario at present, highly suitable habitats increased by 0.05%, while moderately and low-suitable habitats declined (**Figure 6**).

**Figure 7:**
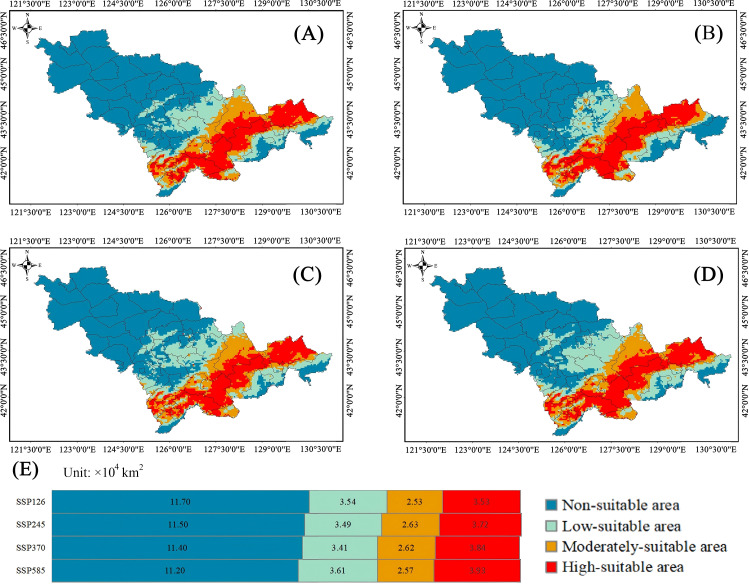
Distribution characteristics of suitable habitat for class II plant resources under future climate scenarios. **(A–D)** Diagram of Plant Area Distribution. **(E)** The chart represents the area.

For Class II plants, in the scenario of SSP 1-2.6, the highly suitable habitats were 3.54×10^4^ km^2^ (16.65%), the moderately suitable habitats were 2.53×10^4^ km^2^ (10.94%) and the low-suitable habitats were 3.53×10^4^ km^2^ (16.60%). Compared with the climate scenario at present, the highly suitable habitats increased by 0.85%. In the scenario of SSP 2-4.5, the highly suitable habitats were 3.49×10^4^ km^2^ (16.42%), the moderately suitable habitats were 2.63×10^4^ km^2^ (12.37%) and the low-suitable habitats were 3.72×10^4^ km^2^ (17.49%). Compared with the climate scenario at present, the highly suitable habitats increased by 0.62%. In the scenario of SSP 3-7.0, the highly suitable habitats were 3.41×10^4^ km^2^ (16.04%), the moderately suitable habitats were 2.62×10^4^ km^2^ (12.32%) and the low-suitable habitats were 3.84×10^4^ km^2^ (18.06%). Compared with the climate scenario at present, the highly suitable habitats, the moderately suitable habitats and low-suitable habitats increased by 0.24%, 0.06% and 0.52%, respectively. In the scenario of SSP 5-8.5, the highly suitable habitats were 3.61×10^4^ km^2^ (16.98%), the moderately suitable habitats were 2.57×10^4^ km^2^ (12.09%) and the low-suitable habitats were 3.93×10^4^ km^2^ (18.48%). Compared with the climate scenario at present, the highly suitable habitats and low-suitable habitats increased by 1.18% and 0.94% (**Figure 7**).

**Figure 8:**
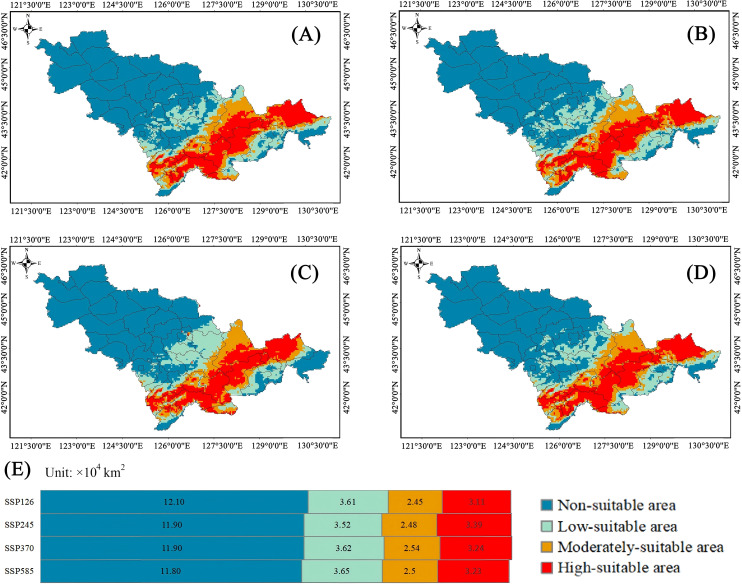
Distribution characteristics of suitable habitat for class III plant resources under future climate scenarios. **(A–D)** Diagram of Plant Area Distribution. **(E)** The chart represents the area.

For Class III plants, in the scenario of SSP 1-2.6, the highly suitable habitats were 3.61×10^4^ km^2^ (16.65%), the moderately suitable habitats were 2.45×10^4^ km^2^ (10.94%) and the low-suitable habitats were 3.11×10^4^ km^2^ (16.60%). In the scenario of SSP 2-4.5, the highly suitable habitats were 3.52×10^4^ km^2^ (16.56%), the moderately suitable habitats were 2.48×10^4^ km^2^ (11.67%) and the low-suitable habitats were 3.39×10^4^ km^2^ (15.95%). Compared with the climate scenario at present, the highly suitable habitats increased. In the scenario of SSP 3-7.0, the highly suitable habitats were 3.62×10^4^ km^2^ (17.03%), the moderately suitable habitats were 2.54×10^4^ km^2^ (11.95%) and the low-suitable habitats were 3.24×10^4^ km^2^ (15.24%). Compared with the climate scenario at present, the moderately suitable habitats increased by 1.37%. In the scenario of SSP 5-8.5, the highly suitable habitats were 3.65×10^4^ km^2^ (17.17%), the moderately suitable habitats were 2.50×10^4^ km^2^ (11.76%) and the low-suitable habitats were 3.23×10^4^ km^2^ (15.19%). Compared with the climate scenario at present, the moderately suitable habitats increased by 1.18% (**Figure 8**).

### 3.5 Model performance

**Figure 9:**
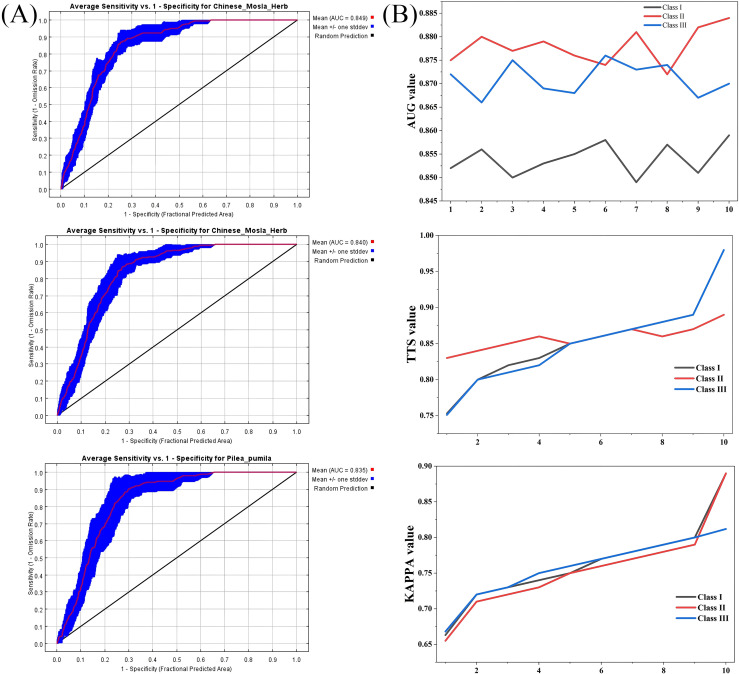
ROC response curve for the MaxEnt model and AUC, TSS and KAPPA values. **(A)** ROC response curve for the MaxEnt model. **(B)** AUC, TSS and KAPPA values of MaxEnt model at different petitions.

The accuracy of MaxEnt model was shown in **Figure 3**. The ROC response curve of MaxEnt model is shown in **Figure 9A**. It was found that the average AUC was 0.849, 0.840, and 0.835, respectively; the average TSS was 0.853, 0.848 and 0.851, respectively; the KAPPA value average was stable at about 0.763, 0.755, 0.768, respectively (**Figure 9B**). The values tend of AUC, TSS and KAPPA was stable in 10 repetitions, which indicated that the MaxEnt had excellent prediction ability and could accurately predict the potential suitable area of Class I - III plant resources.

### 3.6 Migration characteristics for Class II plant resources under future

**Figure 10:**
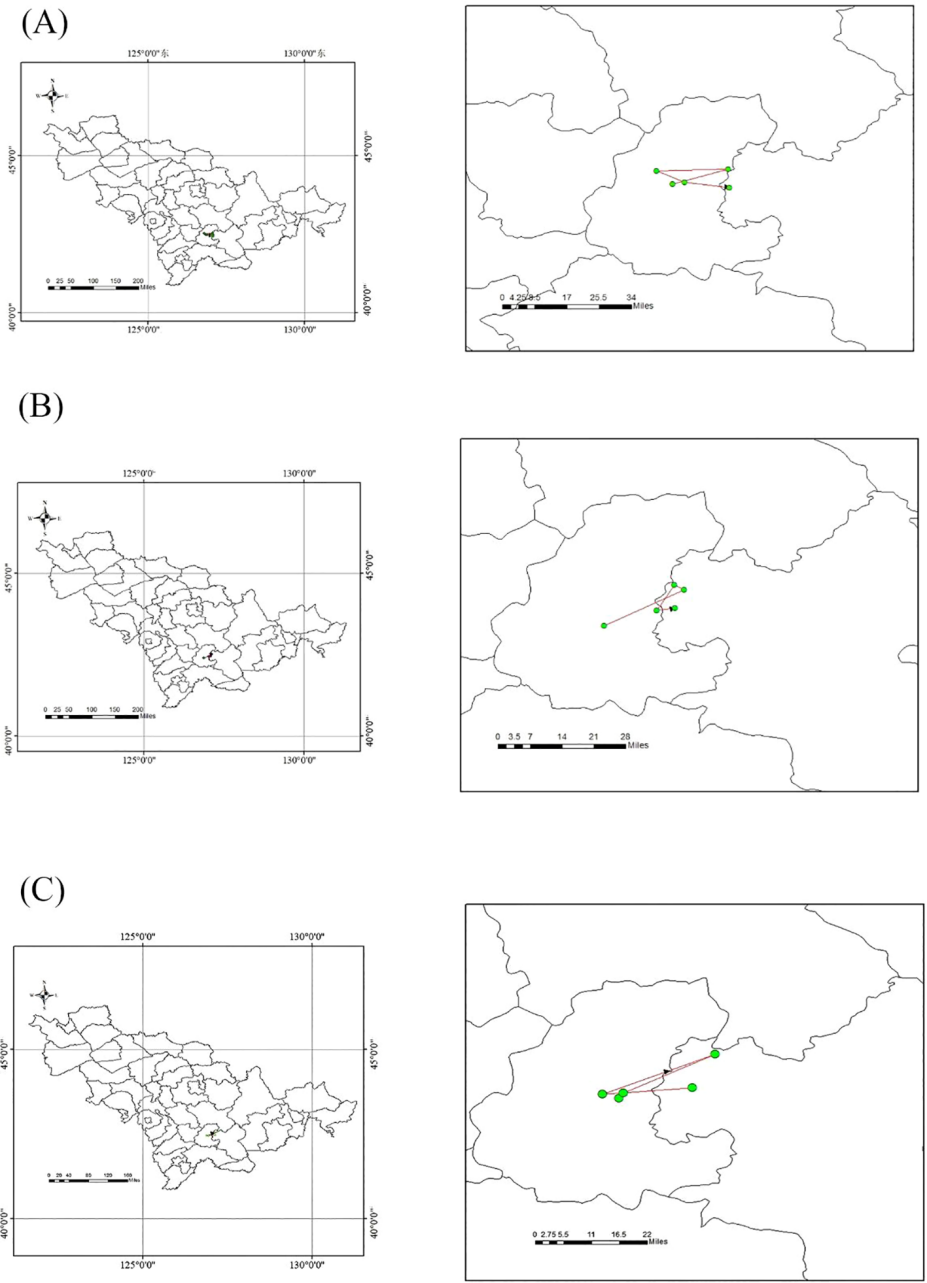
Centroid migration of class I–III resources.

Deeper investigations would focus on their migration patterns under various future scenarios (**Figure 10**). For Class I plant resources, the centroid of suitable habitats shifted 19.43 km towards the northeast under SSP1-2.6, 6.56 km towards the northwest under SSP2-4.5, 4.03 km towards the northeast under SSP3-7.0, and 18.58 km towards the southeast under SSP5-8.5. For Class II plant resources, the centroid shifted 2.13 km towards the northeast under SSP1-2.6, 27.24 km towards the northeast under SSP2-4.5, 4.62 km towards the northwest under SSP3-7.0, and 17.67 km towards the northeast under SSP5-8.5. For Class III plant resources, the centroid moved consistently towards the northeast in all scenarios: 23.91 km under SSP1-2.6, 14.17 km under SSP2-4.5, 14.17 km under SSP3-7.0, and 19.41 km under SSP5-8.5. In summary, the migration centroids of all three classes of medicinal plant resources remained mostly within the Changbai Mountain ecosystem, indicating that the future climate in this region will still support the survival of medicinal plants.

Given the predicted higher developable potential of Class II resources compared to Class I. The high-suitability habitat for Class II plant resources demonstrated consistent expansion in terms of relative growth rate (percentage change in suitable habitat area) under all predicted Shared Socioeconomic Pathway (SSP) scenarios (SSP1-2.6, SSP2-4.5, SSP3-7.0, and SSP5-8.5), reflecting enhanced ecological adaptability under intensified radiative forcing thresholds (**Table 7**). While Class I plant resources remain the current priority for utilization and development due to their established suitability under baseline climatic conditions, this trend indicated a shift: Class II resources were projected to surpass Class I in future exploitable potential.

**Table 7:** Changes in the suitable habitats of class I – III Plant resources under current and future SSP scenarios.

Class	Scenario	Highly suitable (×10^4^ km^2^, %)	Moderately suitable (×10^4^ km^2^, %)	Low-suitable (×10^4^ km^2^, %)	Change relative to present
Class I	SSP 1-2.6	3.38 (15.89)	2.29 (10.77)	3.34 (15.71)	Decreased overall
	SSP 2-4.5	3.61 (16.98)	2.27 (13.03)	3.00 (14.11)	Increased high & moderate
	SSP 3-7.0	3.46 (16.27)	2.38 (15.76)	3.35 (14.11)	Decreased high & low
	SSP 5-8.5	3.49 (16.42)	2.32 (10.94)	3.45 (16.23)	Increased high only
Class II	SSP 1-2.6	3.54 (16.65)	2.53 (10.94)	3.53 (16.60)	Increased high
	SSP 2-4.5	3.49 (16.42)	2.63 (12.37)	3.72 (17.49)	Increased high
	SSP 3-7.0	3.41 (16.04)	2.62 (12.32)	3.84 (18.06)	Increased all three
	SSP 5-8.5	3.61 (16.98)	2.57 (12.09)	3.93 (18.48)	Increased high & low
Class III	SSP 1-2.6	3.61 (16.65)	2.45 (10.94)	3.11 (16.60)	Increased all three
	SSP 2-4.5	3.52 (16.56)	2.48 (11.67)	3.39 (15.95)	Decreased high
	SSP 3-7.0	3.62 (17.03)	2.54 (11.95)	3.24 (15.24)	Decreased high & low
	SSP 5-8.5	3.65 (17.17)	2.50 (11.76)	3.23 (15.19)	Decreased high & low

## 4 Discussion

Most of wild medicinal plant resources have been documented in the Chinese Pharmacopoeia in Changbai Mountain ecosystem (Gu et al., 2024), including *Menispermum dauricum, Elsholtzia ciliata*, and *Platycodon grandiflorus*, and other species. With the growth of market demand for traditional Chinese medicine, supply-demand imbalances have emerged. In this case, it is critical to ensuring stable resource provision for prioritizing exploitation and utilization of these wild medicinal plants (Liu et al., 2024; Wang et al., 2019). Therefore, the assessment of these medicinal plant resources can establish a valuable foundation for prioritizing exploitation, then prediction of these resources can establish a sustainable development for utilization in Changbai Mountain ecosystem.

### 4.1 The rationality of AHP classification

The AHP comprised the target layer, criterion layer, and indicator layer. The criterion and indicator layers were specifically developed based on key botanical and pharmacological resources, including *Flora of China*, *Pharmacopoeia of the People’s Republic of China (2020 edition)*, *Atlas of Medicinal Plant Resources in Northeast China*, *Common Forest Economic Plants of Changbai Mountain*, *List of National Key Protected Wild Plants*, and *Flora of Heilongjiang Province*, ensuring the objectivity of the indicators (**Supplementary Materials Table A.3**). The AHP has been widely applied in relevant research. For instance, Kpadé et al. provided a framework for addressing sustainable forest management challenges in the context of climate change (Kpadé et al., 2024). Similarly, Ramachandra et al. offered rational suggestions for ecosystem protection against land degradation triggered by large-scale land cover changes (Ramachandra et al., 2025). Based on this, our study adopted the AHP for ecosystem conservation and biodiversity protection of 224 wild medicinal plants. Development potential, economic value and endangered status were selected as evaluation standards for the criterion layer (**Figure 2**). Plant resources were divided into three classes (**Table 5**) and their exploitation grades were compared. By consistency validation and matrix operations, it scientifically calculated the weights of influencing elements, thereby enhancing the reliability of decisions (**Table 2**). This classification pattern not only displayed the prioritized utilization of wild medicinal plant resources in the Changbai Mountain ecosystem, but also provided a theoretical basis for the conservation of this ecosystem.

### 4.2 Integration of MaxEnt for evaluation and predictive analysis

Species distribution models play a crucial role in studying ecological issues under the interaction between species and the environment, especially against the backdrop of global climate variables (Dong et al., 2025; Maclean and Early, 2023). The AHP could be applied to analyze plant resources at present, but it was inadequate for suitable habitat analysis and predictions of resource development under future climatic scenarios. MaxEnt is an important aid in understanding the influence of climate change on species distributions. Previous studies had confirmed that the MaxEnt had been widely applied the distribution of species and the impact of climate variables on species. Wang et al. predicted the potential suitable distribution areas of *A. rugosa* at present and future climate scenarios using the MaxEnt and analyzed the dominant climate factors affecting its distribution (Wang et al., 2023); Zhang et al. utilized 374 geographical distribution records of *H. mutabilis* and 19 bioclimatic factors, and then employed the MaxEnt model combined with ArcGIS to analyze the key environmental variables influencing the suitable distribution areas of this species (Zhang et al., 2024). The above studies had provided important methodological and theoretical references for this research. In this study, we constructed three comprehensive models for different categories of medicinal plants (Classes I-III). All 224 species originated from the Changbai Mountain ecosystem and were thus influenced by the same set of major regional environmental driving factors. The integrated habitat suitability area represents the overall suitable habitat for the entire medicinal plant community in the Changbai Mountain region, rather than the actual ecological niche of any single species. The purpose of constructing these unified models was to assess the collective habitat suitability and potential distribution trends of medicinal plant populations, thereby providing scientific basis for regional resource protection and management strategies. The changes and migration characters of suitable habitats across the Classes I-III were analyzed by integrating the MaxEnt with ArcGIS (**Figure 2**), the characteristics of priority utilization were intensively studied under future climatic scenarios, and key climate impact factors were identified. The suitable habitats of Class II plants under future climatic scenarios did not shrink but rather expanded, exhibiting a shift towards the northeast and northwest, and exhibited habitat expansion and migration (Hu et al., 2015; Salgotra and Chauhan, 2023) (**Figures 7** and **9**). The research by Feldman et al. showed that habitat expansion and migration indicated enhanced climatic suitability for plants under future climatic scenarios (Feldman et al., 2024). Therefore, although Class I plants were currently the most suitable for utilization, they face habitat shrinkage under most future climate scenarios.

Climate change would bring a distinct threat to biodiversity, resulting in shrinkage of habitat areas and extinction risk (Veldkornet, 2023; Koc et al., 2024). Previous studies have shown that the physiological processes and distribution patterns of plants are influenced by climatic factors such as temperature and precipitation. The research by Girón-Gutiérrez et al. found that the temperature variable had the highest correlation with above-ground biomass density (76%), and this correlation showed significant interspecific differences; while the relationship between precipitation factors and above-ground biomass density was mainly positive. Specifically, precipitation has a significant impact on plant distribution. The article by Li et al. pointed out that the optimal model included five climatic variables, among which the precipitation factor played an important role. This indicates that in climate models, precipitation is a key factor affecting plant distribution (Girón-Gutiérrez et al., 2024; Li et al., 2020). Bernacchi et al. in their article discussed the adaptability of plant biochemistry and metabolism induced by temperature, and these adaptations affect photosynthesis and biomass production (Bernacchi et al., 2023). In our study, the MaxEnt consistently identified Bio11 and Bio12 as the dominant drivers of habitat suitability across all classes. In terms of conservation, this indicates that protected areas are likely to diminish in effectiveness over time (Foyer and Kranner, 2023; Leisner et al., 2023). Based on climate variables, we studied the future climate scenarios suitable habitats of wild medicinal plant resources which provided a foundation for the conservation of biodiversity and the prioritized development and utilization of resources.

Although this study provides valuable insights into the prioritized development of wild medicinal plants in the Changbai Mountain ecosystem, habitat prediction, and conservation strategies, it must be acknowledged that there are some limitations. These limitations offer directions for future related research. Firstly, although we conducted field investigations and improved spatial clustering based on the data, there are still potential limitations and uncertainties in the field sampling. Based on the collected distribution data, the study area may still be affected to varying degrees by transportation inconvenience, uneven sampling work, and complex terrain conditions. Moreover, the records of some individual species are relatively few (<20), and we acknowledge that the small sample size of certain species may still introduce small uncertainties. These factors may lead to sampling bias and affect the accuracy and reliability of the habitat suitability modeling results. Secondly, this study only relies on a global climate model to predict future habitat conditions, which will inevitably bring additional uncertainty to the simulation results. The variability and structural differences of different climate models are not considered here, which may limit the robustness of future predictions. Thirdly, to exclude scattered, marginal and temporarily recorded locations, we only retained the locations near the center of each species’ distribution. Although this processing method aims to improve data quality, it may lead to spatial clustering of distribution points, which may further interfere with the performance of the MaxEnt model. Specifically, this approach may exacerbate sample bias, causing the model to overestimate the suitability of habitats in densely sampled areas and underestimate it in surrounding or under-sampled areas, thereby damaging the overall reliability of the data set and the accuracy of the modeling results. When interpreting the results of this study, one should carefully consider these limitations, and they can provide meaningful guidance and direction for the further improvement of related future research.

### 4.3 Conservation strategies and management implications

According to the research results, the targeted protection and management strategies of wild medicinal plants in Changbai Mountain ecosystem were put forward. First of all, Class I medicinal plants have high development value, but it faces habitat shrinkage in the future climate scenario. Over-exploitation should be limited to prevent further degradation of its suitable habitats. Secondly, in view of the habitat expansion of Class II medicinal plants in the future climate scenario, these species can be regarded as priority candidates for sustainable utilization and artificial cultivation. Thirdly, considering that Bio11 (Mean temperature of coldest quarter), Bio12 (Annual precipitation) are the main climate drivers, long-term monitoring of temperature and precipitation dynamics should be strengthened to support adaptive protection planning under climate change. Fourthly, in order to reduce sampling bias and model uncertainty in future research, a standardized field survey network should be established to improve the quantity and spatial balance of species distribution records, and a set of multi-climate models should be adopted to improve the reliability of habitat prediction. The integration of AHP and MaxEnt model used in this study can be used as a scientific framework to guide the rational development, effective protection and long-term sustainable management of wild medicinal plant resources in Changbai Mountain ecosystem.

## 5 Conclusion

In the study, 224 medicinal plant resources in the Changbai Mountains were classified into three categories via the analytic hierarchy process (AHP). Meanwhile, by using the optimized MaxEnt model and integrating climatic variables, we predicted the potentially suitable habitats of wild medicinal plant resources at present and future climate scenarios. The AUC, TSS and KAPPA values from the model outputs demonstrated that the model accurately simulates the distribution of wild medicinal plant resources. Among the climate variables, mean temperature of the coldest quarter (Bio11) and annual precipitation (Bio12) significantly influence the plants’ distribution. Crucially, while Class I were most suitable for utilization under the present climatic scenario, our predictions revealed that Class II would surpass them under future climate scenarios. These findings enhanced the understanding of the potential distribution patterns of wild medicinal plant resources and provided a scientific basis for prioritizing their utilization and conserving the biodiversity of medicinal plant resources.

## Data Availability

The original contributions presented in the study are included in the article/**Supplementary Material**. Further inquiries can be directed to the corresponding authors.
